# 1-[(3-Chloro­phen­yl)(morpholin-4-yl)­meth­yl]naphthalen-2-ol

**DOI:** 10.1107/S1600536811021726

**Published:** 2011-06-18

**Authors:** Li-Jin Shu

**Affiliations:** a Key Laboratory of Organosilicon Chemistry and Material Technology of the Ministry of Education, Hangzhou Normal University, Hangzhou 310012, People’s Republic of China

## Abstract

In the title compound, C_21_H_20_ClNO_2_, the dihedral angle between the naphthyl­ene ring system and the phenyl ring is 77.86 (15)°. The morpholine ring adopts a chair conformation. The hydroxyl group is involved in intra­molecular O—H⋯N hydrogen bonding. A weak inter­molecular C—H⋯π inter­action is present in the crystal structure.

## Related literature

For related structures, see: Devi & Bhuyan (2004 ▶); Domling & Ugi (2000 ▶); Fu *et al.* (2009 ▶). For multi-component reactions, see: Hulme & Gore (2003 ▶); Ugi (1962 ▶).
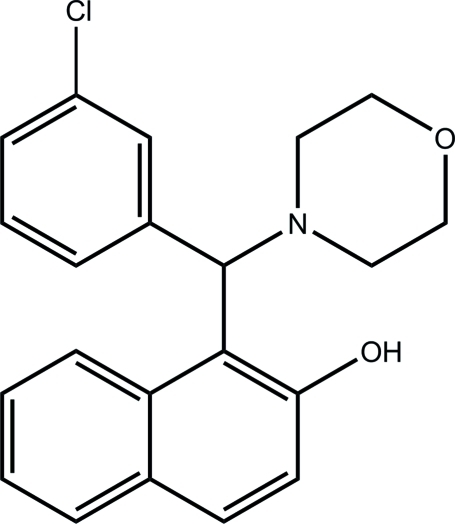


## Experimental

### 

#### Crystal data

C_21_H_20_ClNO_2_
*M* *_r_* = 353.83
Monoclinic, 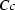

*a* = 14.1896 (18) Å
*b* = 15.881 (2) Å
*c* = 10.3998 (10) Å
β = 132.13 (2)°
*V* = 1738.0 (7) Å^3^
*Z* = 4
Mo *K*α radiation
μ = 0.23 mm^−1^
*T* = 298 K
0.40 × 0.30 × 0.20 mm

#### Data collection

Rigaku Mercury2 diffractometer
Absorption correction: multi-scan (*CrystalClear*; Rigaku, 2005 ▶) *T*_min_ = 0.89, *T*_max_ = 1.00
7216 measured reflections
3127 independent reflections
2050 reflections with *I* > 2σ(*I*)
*R*_int_ = 0.105

#### Refinement

*R*[*F* ^2^ > 2σ(*F* ^2^)] = 0.085
*wR*(*F* ^2^) = 0.240
*S* = 0.99
3127 reflections
226 parameters
2 restraints
H-atom parameters constrained
Δρ_max_ = 0.33 e Å^−3^
Δρ_min_ = −0.29 e Å^−3^
Absolute structure: Flack (1983 ▶), 1551 Friedel pairs
Flack parameter: 0.07 (16)

### 

Data collection: *CrystalClear* (Rigaku, 2005 ▶); cell refinement: *CrystalClear*; data reduction: *CrystalClear*; program(s) used to solve structure: *SHELXTL* (Sheldrick, 2008 ▶); program(s) used to refine structure: *SHELXTL*; molecular graphics: *SHELXTL*; software used to prepare material for publication: *SHELXTL*.

## Supplementary Material

Crystal structure: contains datablock(s) I, global. DOI: 10.1107/S1600536811021726/xu5238sup1.cif

Structure factors: contains datablock(s) I. DOI: 10.1107/S1600536811021726/xu5238Isup2.hkl

Additional supplementary materials:  crystallographic information; 3D view; checkCIF report

## Figures and Tables

**Table 1:** Hydrogen-bond geometry (Å, °) *Cg* is the centroid of the C3–C8 benzene ring.

*D*—H⋯*A*	*D*—H	H⋯*A*	*D*⋯*A*	*D*—H⋯*A*
O1—H1*A*⋯N1	0.82	1.97	2.649 (6)	139
C21—H21*A*⋯*Cg*^i^	0.93	2.87	3.770 (7)	164

Symmetry code: (i) 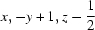.

**Fig. 1.**
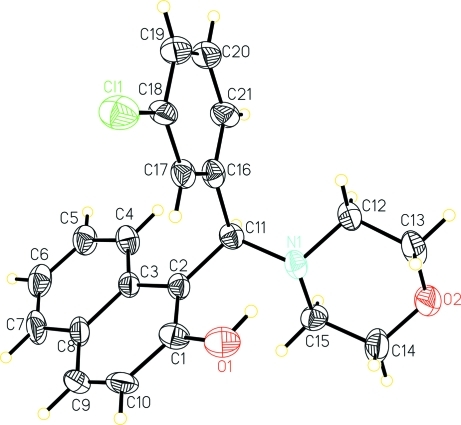
A view of the asymmetric unit with the atomic numbering scheme. The displacement ellipsoids were drawn at the 30% probability level.

## supplementary crystallographic information

### Comment

Multi-component reactions (MCRs) (Hulme & Gore, 2003; Ugi, 1962) involving at least three starting materials in a one-pot reaction have attracted considerable attention in terms of saving both energy and raw materials (Devi & Bhuyan, 2004; Fu, *et al.* 2009). Compared to conventional multi-step organic syntheses, MCRs have merits over multi-step reactions that include the simplicity of a one-pot procedure and the buildup of complex molecules (Domling & Ugi, 2000). We report here the synthesis and crystal structure of the title compound, 1-((3-chlorophenyl)(morpholino)methyl)naphthalen-2-ol.

In the title compound (Fig. 1) bond lengths and angles have normal values. The dihedral angle between the naphthylene ring system and the benzene ring is 77.86 (15)°. The H atom of hydroxyl is involved in strong intramolecular O—H···N hydrogen bonds (Table 1). Weak intermolecular C—H···π interaction is present in the crystal structure.

### Experimental

A dry 50 ml flask was charged with 3-chlorobenzaldehyde (10 mmol), naphthalen-2-ol (10 mmol) and morpholine (10 mmol). The mixture was stirred at 373 K for 12 h and then added ethanol (15 ml), after heated under reflux for 1 h, the precipitate was filtered out and washed with ethanol for 3 times to give the title compound. Colourless crystals suitable for X-ray diffraction were obtained by slow evaporation of a dichloromethane solution.

### Refinement

All the H atoms attached to C atoms were situated into the idealized positions and treated as riding with C–H = 0.93 Å (aromatic), 0.97 Å (methylene) and 0.98 Å (methine) with *U_iso_*(H)=1.2*U_eq_*(C). The positional parameters of the H atom (O1) was refined freely. And in the last stage of the refinement, it was restrained with the H1—O1 = 0.82 (2)Å), with *U_iso_*(H)=1.5*U_eq_*(O).

### Crystal data

**Table:**

C_21_H_20_ClNO_2_	*F*(000) = 744
*M**_r_* = 353.83	*D*_x_ = 1.352 Mg m^−^^3^
Monoclinic, *C**c*	Mo *K*α radiation, λ = 0.71073 Å
Hall symbol: C -2yc	Cell parameters from 3951 reflections
*a* = 14.1896 (18) Å	θ = 3.7–27.5°
*b* = 15.881 (2) Å	µ = 0.23 mm^−^^1^
*c* = 10.3998 (10) Å	*T* = 298 K
β = 132.13 (2)°	Block, colourless
*V* = 1738.0 (7) Å^3^	0.40 × 0.30 × 0.20 mm
*Z* = 4	

### Data collection

**Table:**

Rigaku Mercury2 diffractometer	3127 independent reflections
Radiation source: fine-focus sealed tube	2050 reflections with *I* > 2σ(*I*)
graphite	*R*_int_ = 0.105
Detector resolution: 13.6612 pixels mm^-1^	θ_max_ = 25.2°, θ_min_ = 3.7°
CCD profile fitting scans	*h* = −16→16
Absorption correction: multi-scan (*CrystalClear*; Rigaku, 2005)	*k* = −18→18
*T*_min_ = 0.89, *T*_max_ = 1.00	*l* = −12→12
7216 measured reflections	

### Refinement

**Table:**

Refinement on *F*^2^	Secondary atom site location: difference Fourier map
Least-squares matrix: full	Hydrogen site location: inferred from neighbouring sites
*R*[*F*^2^ > 2σ(*F*^2^)] = 0.085	H-atom parameters constrained
*wR*(*F*^2^) = 0.240	*w* = 1/[σ^2^(*F*_o_^2^) + (0.116*P*)^2^ + 0.730*P*] where *P* = (*F*_o_^2^ + 2*F*_c_^2^)/3
*S* = 0.99	(Δ/σ)_max_ < 0.001
3127 reflections	Δρ_max_ = 0.33 e Å^−^^3^
226 parameters	Δρ_min_ = −0.29 e Å^−^^3^
2 restraints	Absolute structure: Flack (1983), 1551 Friedel pairs
Primary atom site location: structure-invariant direct methods	Flack parameter: 0.07 (16)

### Special details

**Table:**

Geometry. All esds (except the esd in the dihedral angle between two l.s. planes) are estimated using the full covariance matrix. The cell esds are taken into account individually in the estimation of esds in distances, angles and torsion angles; correlations between esds in cell parameters are only used when they are defined by crystal symmetry. An approximate (isotropic) treatment of cell esds is used for estimating esds involving l.s. planes.
Refinement. Refinement of F^2^ against ALL reflections. The weighted R-factor wR and goodness of fit S are based on F^2^, conventional R-factors R are based on F, with F set to zero for negative F^2^. The threshold expression of F^2^ > 2sigma(F^2^) is used only for calculating R-factors(gt) etc. and is not relevant to the choice of reflections for refinement. R-factors based on F^2^ are statistically about twice as large as those based on F, and R- factors based on ALL data will be even larger.

### Fractional atomic coordinates and isotropic or equivalent isotropic displacement parameters (Å^2^)

**Table:**

	*x*	*y*	*z*	*U*_iso_*/*U*_eq_	
Cl1	0.84978 (14)	0.53891 (11)	0.59226 (19)	0.0747 (5)	
N1	0.4387 (4)	0.6957 (3)	−0.0609 (6)	0.0481 (11)	
C4	0.2270 (5)	0.5435 (4)	0.0113 (7)	0.0554 (15)	
H4A	0.2462	0.5206	−0.0511	0.066*	
C2	0.3791 (4)	0.6606 (3)	0.1069 (6)	0.0433 (13)	
C3	0.2871 (4)	0.6169 (4)	0.1035 (7)	0.0458 (12)	
O1	0.5267 (4)	0.7746 (2)	0.2256 (5)	0.0681 (12)	
H1A	0.5381	0.7522	0.1660	0.102*	
C10	0.4042 (6)	0.7604 (4)	0.3021 (8)	0.0615 (17)	
H10A	0.4455	0.8085	0.3690	0.074*	
C11	0.4105 (4)	0.6255 (4)	0.0053 (6)	0.0441 (13)	
H11A	0.3346	0.5962	−0.0957	0.053*	
C18	0.7153 (5)	0.5175 (4)	0.3731 (7)	0.0506 (13)	
C1	0.4344 (5)	0.7309 (4)	0.2081 (7)	0.0530 (15)	
C17	0.6232 (4)	0.5757 (4)	0.2811 (7)	0.0496 (14)	
H17A	0.6314	0.6259	0.3337	0.060*	
O2	0.3993 (4)	0.7915 (3)	−0.3232 (6)	0.0786 (13)	
C16	0.5163 (5)	0.5618 (4)	0.1089 (6)	0.0474 (13)	
C21	0.5104 (5)	0.4884 (3)	0.0364 (8)	0.0536 (15)	
H21A	0.4409	0.4773	−0.0798	0.064*	
C6	0.1102 (6)	0.5319 (5)	0.0997 (8)	0.071 (2)	
H6A	0.0540	0.5025	0.1006	0.085*	
C15	0.3269 (5)	0.7510 (4)	−0.1813 (8)	0.0530 (14)	
H15A	0.2571	0.7192	−0.2820	0.064*	
H15B	0.2997	0.7736	−0.1241	0.064*	
C12	0.4812 (5)	0.6659 (4)	−0.1490 (7)	0.0529 (14)	
H12A	0.5596	0.6343	−0.0681	0.063*	
H12B	0.4175	0.6288	−0.2439	0.063*	
C8	0.2555 (5)	0.6503 (4)	0.1990 (7)	0.0578 (16)	
C19	0.7135 (6)	0.4438 (4)	0.3062 (8)	0.0629 (16)	
H19A	0.7796	0.4051	0.3719	0.075*	
C20	0.6086 (7)	0.4297 (4)	0.1365 (9)	0.0699 (17)	
H20A	0.6023	0.3794	0.0856	0.084*	
C9	0.3224 (6)	0.7255 (4)	0.3030 (8)	0.0646 (16)	
H9A	0.3065	0.7482	0.3693	0.077*	
C7	0.1665 (6)	0.6075 (6)	0.1928 (9)	0.080 (2)	
H7A	0.1439	0.6296	0.2518	0.096*	
C14	0.3634 (7)	0.8196 (5)	−0.2346 (9)	0.083 (2)	
H14A	0.2923	0.8581	−0.3084	0.099*	
H14B	0.4334	0.8506	−0.1325	0.099*	
C13	0.5017 (6)	0.7377 (5)	−0.2158 (9)	0.074 (2)	
H13A	0.5733	0.7699	−0.1181	0.089*	
H13B	0.5247	0.7165	−0.2790	0.089*	
C5	0.1409 (6)	0.5033 (5)	0.0082 (8)	0.0641 (17)	
H5A	0.1017	0.4548	−0.0583	0.077*	

### Atomic displacement parameters (Å^2^)

**Table:**

	*U*^11^	*U*^22^	*U*^33^	*U*^12^	*U*^13^	*U*^23^
Cl1	0.0583 (8)	0.0818 (10)	0.0641 (8)	0.0128 (8)	0.0329 (7)	−0.0010 (8)
N1	0.0500 (19)	0.057 (3)	0.050 (2)	−0.0125 (19)	0.0387 (17)	−0.007 (2)
C4	0.046 (2)	0.075 (4)	0.051 (3)	−0.003 (3)	0.035 (2)	0.002 (3)
C2	0.040 (2)	0.053 (3)	0.035 (2)	0.005 (2)	0.025 (2)	0.008 (2)
C3	0.045 (2)	0.056 (3)	0.043 (2)	0.011 (2)	0.033 (2)	0.012 (2)
O1	0.066 (2)	0.058 (2)	0.070 (2)	−0.005 (2)	0.042 (2)	−0.010 (2)
C10	0.073 (3)	0.047 (3)	0.056 (3)	0.013 (3)	0.040 (3)	−0.008 (3)
C11	0.035 (2)	0.055 (3)	0.041 (2)	0.002 (2)	0.0247 (19)	0.001 (2)
C18	0.058 (3)	0.047 (3)	0.066 (3)	0.008 (2)	0.050 (2)	0.000 (2)
C1	0.048 (3)	0.052 (3)	0.051 (3)	−0.003 (3)	0.030 (2)	−0.013 (3)
C17	0.042 (2)	0.066 (4)	0.053 (3)	0.005 (2)	0.036 (2)	0.008 (3)
O2	0.105 (3)	0.081 (3)	0.077 (2)	0.036 (2)	0.072 (2)	0.033 (2)
C16	0.052 (2)	0.053 (3)	0.050 (3)	−0.016 (2)	0.039 (2)	−0.009 (2)
C21	0.064 (3)	0.044 (3)	0.058 (3)	−0.005 (3)	0.043 (3)	−0.013 (3)
C6	0.061 (3)	0.090 (5)	0.074 (4)	0.009 (3)	0.050 (3)	0.026 (4)
C15	0.046 (3)	0.052 (3)	0.055 (3)	0.010 (2)	0.031 (2)	0.009 (3)
C12	0.059 (3)	0.062 (3)	0.052 (3)	0.000 (3)	0.043 (2)	0.000 (3)
C8	0.064 (3)	0.077 (4)	0.049 (3)	0.018 (3)	0.045 (2)	0.024 (3)
C19	0.085 (3)	0.043 (3)	0.075 (3)	−0.001 (3)	0.059 (3)	−0.007 (3)
C20	0.108 (4)	0.040 (3)	0.096 (4)	−0.002 (3)	0.083 (3)	−0.004 (3)
C9	0.094 (3)	0.057 (4)	0.062 (3)	0.024 (3)	0.060 (3)	0.006 (3)
C7	0.083 (3)	0.122 (6)	0.078 (3)	0.031 (4)	0.071 (3)	0.037 (4)
C14	0.095 (4)	0.097 (5)	0.090 (4)	0.033 (4)	0.075 (3)	0.045 (4)
C13	0.084 (3)	0.096 (5)	0.077 (4)	−0.004 (4)	0.068 (3)	−0.001 (4)
C5	0.061 (3)	0.074 (4)	0.070 (4)	−0.003 (3)	0.050 (3)	0.007 (3)

### Geometric parameters (Å, °)

**Table:**

Cl1—C18	1.765 (6)	C16—C21	1.360 (8)
N1—C12	1.472 (7)	C21—C20	1.395 (9)
N1—C15	1.480 (7)	C21—H21A	0.9300
N1—C11	1.499 (7)	C6—C5	1.362 (10)
C4—C5	1.359 (9)	C6—C7	1.407 (11)
C4—C3	1.382 (8)	C6—H6A	0.9300
C4—H4A	0.9300	C15—C14	1.465 (10)
C2—C1	1.364 (7)	C15—H15A	0.9700
C2—C3	1.459 (7)	C15—H15B	0.9700
C2—C11	1.502 (8)	C12—C13	1.462 (10)
C3—C8	1.437 (8)	C12—H12A	0.9700
O1—C1	1.381 (7)	C12—H12B	0.9700
O1—H1A	0.8200	C8—C7	1.396 (9)
C10—C9	1.290 (9)	C8—C9	1.457 (9)
C10—C1	1.387 (9)	C19—C20	1.362 (9)
C10—H10A	0.9300	C19—H19A	0.9300
C11—C16	1.506 (7)	C20—H20A	0.9300
C11—H11A	0.9800	C9—H9A	0.9300
C18—C17	1.342 (7)	C7—H7A	0.9300
C18—C19	1.353 (8)	C14—H14A	0.9700
C17—C16	1.383 (7)	C14—H14B	0.9700
C17—H17A	0.9300	C13—H13A	0.9700
O2—C13	1.383 (8)	C13—H13B	0.9700
O2—C14	1.393 (9)	C5—H5A	0.9300
			
C12—N1—C15	108.6 (5)	C14—C15—H15A	110.1
C12—N1—C11	113.2 (4)	N1—C15—H15A	110.1
C15—N1—C11	111.4 (4)	C14—C15—H15B	110.1
C5—C4—C3	122.7 (7)	N1—C15—H15B	110.1
C5—C4—H4A	118.7	H15A—C15—H15B	108.4
C3—C4—H4A	118.7	C13—C12—N1	109.9 (5)
C1—C2—C3	116.8 (5)	C13—C12—H12A	109.7
C1—C2—C11	124.0 (5)	N1—C12—H12A	109.7
C3—C2—C11	119.2 (5)	C13—C12—H12B	109.7
C4—C3—C8	117.2 (5)	N1—C12—H12B	109.7
C4—C3—C2	123.0 (5)	H12A—C12—H12B	108.2
C8—C3—C2	119.8 (5)	C7—C8—C3	118.7 (6)
C1—O1—H1A	109.5	C7—C8—C9	123.5 (6)
C9—C10—C1	124.4 (6)	C3—C8—C9	117.7 (5)
C9—C10—H10A	117.8	C18—C19—C20	115.8 (6)
C1—C10—H10A	117.8	C18—C19—H19A	122.1
N1—C11—C2	110.0 (4)	C20—C19—H19A	122.1
N1—C11—C16	112.5 (4)	C19—C20—C21	122.3 (6)
C2—C11—C16	111.7 (4)	C19—C20—H20A	118.9
N1—C11—H11A	107.4	C21—C20—H20A	118.9
C2—C11—H11A	107.4	C10—C9—C8	119.2 (6)
C16—C11—H11A	107.4	C10—C9—H9A	120.4
C17—C18—C19	123.7 (6)	C8—C9—H9A	120.4
C17—C18—Cl1	118.7 (5)	C8—C7—C6	121.7 (7)
C19—C18—Cl1	117.5 (4)	C8—C7—H7A	119.2
C2—C1—O1	121.1 (6)	C6—C7—H7A	119.2
C2—C1—C10	122.0 (6)	O2—C14—C15	113.0 (7)
O1—C1—C10	116.8 (5)	O2—C14—H14A	109.0
C18—C17—C16	120.8 (6)	C15—C14—H14A	109.0
C18—C17—H17A	119.6	O2—C14—H14B	109.0
C16—C17—H17A	119.6	C15—C14—H14B	109.0
C13—O2—C14	108.4 (5)	H14A—C14—H14B	107.8
C21—C16—C17	117.4 (5)	O2—C13—C12	115.5 (5)
C21—C16—C11	121.2 (5)	O2—C13—H13A	108.4
C17—C16—C11	121.4 (5)	C12—C13—H13A	108.4
C16—C21—C20	119.9 (5)	O2—C13—H13B	108.4
C16—C21—H21A	120.0	C12—C13—H13B	108.4
C20—C21—H21A	120.0	H13A—C13—H13B	107.5
C5—C6—C7	117.7 (6)	C4—C5—C6	122.0 (7)
C5—C6—H6A	121.1	C4—C5—H5A	119.0
C7—C6—H6A	121.1	C6—C5—H5A	119.0
C14—C15—N1	108.2 (5)		

### Hydrogen-bond geometry (Å, °)

**Table:**

Cg is the centroid of the C3–C8 benzene ring.

**Table:**

*D*—H···*A*	*D*—H	H···*A*	*D*···*A*	*D*—H···*A*
O1—H1A···N1	0.82	1.97	2.649 (6)	139
C21—H21A···Cg^i^	0.93	2.87	3.770 (7)	164

Symmetry codes: (i) *x*, −*y*+1, *z*−1/2.

## References

[bb1] Devi, I. & Bhuyan, P. J. (2004). *Tetrahedron Lett.* **45**, 8625–8627.

[bb2] Domling, A. & Ugi, I. (2000). *Angew. Chem. Int. Ed.* **39**, 3168–3210. 10.1002/1521-3773(20000915)39:18<3168::aid-anie3168>3.0.co;2-u 11028061

[bb3] Flack, H. D. (1983). *Acta Cryst.* A**39**, 876–881.

[bb4] Fu, D.-W., Ge, J.-Z., Dai, J., Ye, H.-Y. & Qu, Z.-R. (2009). *Inorg. Chem. Commun.* **12**, 994-997.

[bb5] Hulme, C. & Gore, V. (2003). *Curr. Med. Chem.* **10**, 51–8. 10.2174/0929867033368600 12570721

[bb6] Rigaku (2005). *CrystalClear* Rigaku Corporation, Tokyo, Japan.

[bb7] Sheldrick, G. M. (2008). *Acta Cryst.* A**64**, 112–122. 10.1107/S0108767307043930 18156677

[bb8] Ugi, I. (1962). *Angew. Chem. Int. Ed. Engl.* **1**, 8–21.

